# The Value of First-Generation College Students in Medicine: Bridging Gaps and Driving Innovation

**DOI:** 10.7759/cureus.83912

**Published:** 2025-05-11

**Authors:** Emilie A Foltz, Alexander J Pursel, Leila Harrison

**Affiliations:** 1 School of Medicine, Washington State University, Spokane, USA; 2 School of Medicine, Washington State University, Vancouver, USA

**Keywords:** first-generation, first-generation students, graduate medical education (gme), higher education medical training, undergraduate and graduate medical education

## Abstract

First-generation college students in medicine are crucial to advancing health equity and innovation in the evolving medical field. These students, often the first in their extended families to graduate, bring diverse perspectives, resilience, and a strong commitment to underserved communities. Their unique backgrounds enhance patient care, foster innovation, and help address physician shortages in underserved areas. However, they face challenges such as financial constraints and lack of mentorship. This paper underscores the need for institutional support systems to help these students succeed. Ultimately, their inclusion is essential for creating a more equitable and compassionate healthcare system, making their development a strategic imperative for the future of medicine.

## Editorial

Introduction

The landscape of modern medicine is constantly evolving, shaped by advances in technology, shifts in patient demographics, and the ongoing pursuit of health equity. Amidst these changes, one group has emerged as a critical catalyst for progress: first-generation college students entering the field of medicine. These individuals, who are the first in their families to graduate from college, bring unique perspectives and lived experiences, resilience, and a deep commitment to serving their communities.

This editorial explores the value of first-generation medical students, drawing on recent research to highlight their contributions and the challenges they face.

Diverse perspectives and enhanced patient care

First-generation college students often come from diverse backgrounds, bringing a wealth of experiences and perspectives that enrich the medical field. It has been shown that diversity in medical teams leads to improved patient outcomes and satisfaction [1]. Diversity is not limited to race or ethnicity, but also includes socioeconomic background, geographic origin, and life experiences [2]. First-generation students, who may have faced significant barriers to higher education, are particularly attuned to the needs of underserved and marginalized communities [3].

Their firsthand understanding of these challenges allows them to connect with patients on a deeper level, fostering trust and improving communication. It has been proposed that patients from underrepresented backgrounds may have higher satisfaction with medical care when treated by physicians who understand their cultural and socioeconomic contexts [4]. This connection is crucial in addressing health disparities and ensuring all patients receive equitable care.

Resilience and problem-solving skills

First-generation college students often exhibit remarkable resilience, having navigated complex educational and financial landscapes and barriers to achieve their goals. This resilience translates into the medical field where the ability to overcome obstacles is essential. Research indicates that first-generation medical students are adept at problem-solving and resourcefulness, skills that are invaluable in clinical settings [3,5].

These students are often “self-starters” who have become proficient at advocating for themselves and others making them effective leaders and collaborators [6]. Their ability to persevere in the face of adversity prepares them well for the rigors of medical training and practice. Furthermore, their experiences can inspire peers and colleagues, fostering a culture of perseverance and innovation within medical institutions.

Addressing physician shortages in underserved areas

One of the most pressing issues in healthcare today is the shortage of physicians in underserved areas. First-generation medical students are uniquely positioned to address this gap. Many of these students come from rural or economically disadvantaged regions and feel a strong sense of duty to return and serve their communities [6,7].

This trend is vital in mitigating healthcare disparities and ensuring that all populations have access to quality care. By returning to their roots, first-generation physicians not only address immediate healthcare needs but can also serve as role models, inspiring future generations to pursue careers in medicine [3].

Innovative approaches to medical education and practice

First-generation students often bring innovative approaches to medical education and practice, challenging traditional norms and fostering new ideas. Their unique perspectives can drive curriculum changes that better reflect the needs of diverse populations [5,8,9].

Given their unique experiences, these students can champion the integration of community-based learning and service into medical education, promoting a more holistic approach to healthcare [6]. Their advocacy for patient-centered care and community engagement is crucial in developing a medical workforce that is responsive to the evolving needs of society.

Challenges and support systems

Despite their valuable contributions, first-generation medical students face significant challenges, including financial constraints, lack of mentorship, and the pressure to balance family expectations with academic demands. Christophers et al. identified that financial stress was a particularly major barrier for these students, often leading to increased anxiety and burnout [5,6].

There are also significant systematic barriers that impede first-generation students' entry into medical school. They may experience issues of access or lack the social capital to be prepared to submit applications that meet the traditional expectation of what makes a “competitive” applicant. Appel (2023) challenges the admissions community to reconsider how and what is viewed as “intelligence” to achieve acceptance into medical school [9]. Traditional admissions frameworks which prioritized academic metrics over other equally important factors such as life experiences and character traits may be leaving first-generation applicants behind [8]. In a comprehensive national study across four admissions cycles, first-generation applicants had higher levels of non-medical and medical employment, but had fewer honors and awards, shadowing, and community service [9]. In the end, the latter experiences had greater positive associations with acceptance than employment [9]. If greater numbers of first-generation students are working, whether due to obligations to support themselves or others, and employment is devalued over other traditional activities, these students are inherently disadvantaged in admissions.

There are examples of medical schools which serve as models in how they consider the value first-generation and students from other broadly diverse backgrounds bring to medical education. Nguyen et al. describe several tools and strategies for more holistically reviewing applicants to health professions programs, including medicine. These include setting thresholds for academic metrics, using the socioeconomic disadvantaged score (SED) developed by the University of California Davis School of Medicine, and ensuring those involved in making admissions decisions are also a broadly diverse group of people as well as trained fully in the admissions process and bias mitigation [10]. Our own medical school has implemented a thoughtful and intentional holistic admissions process that masks academic metrics (i.e., scores on the Medical College Admission Test [MCAT] and grade point averages [GPA]) once a threshold combination has been met at the pre-screening stage [11]. This has resulted in increased representation of mission-aligned groups, including first-generation college students, as admissions decisions are made throughout any given cycle [12]. Table 1 shows the representation of first-generation students at our medical school compared to national numbers. These are proven strategies that can help other medical schools reconsider how to use their agency to break down structural barriers for first-generation students.

**Table 1:** Percentage of Applied, Accepted, and Matriculated First-Generation Medical Students at the Washington State University Elson S. Floyd College of Medicine and nationally at Association of American Medical Colleges Schools (AAMC FACTS) from Entering Year 2020 to 2023. Source: AAMC FACTS (https://www.aamc.org/media/66851/download?attachment)

Status	Entering Year	WSU no. (%)	National no. (%)
Applied	2020	275 (19%)	7,421 (14%)
	2021	392 (22.3%)	9,616 (15.4%)
	2022	288 (20%)	7,712 (14%)
	2023	334 (20.7%)	7,260 (13.8%)
Accepted	2020	42 (27.1%)	2,647 (11.5%)
	2021	42 (31.3%)	2,928 (12.3%)
	2022	37 (28%)	2,667 (11.2%)
	2023	28 (21.2%)	2,720 (11.3%)
Matriculated	2020	27 (33.8%)	2,545 (11.4%)
	2021	29 (36.3%)	2,804 (12.4%)
	2022	26 (32.5%)	2,543 (11.2%)
	2023	17 (21.3%)	2,606 (11.3%)

To support first-generation medical students, institutions must develop robust support systems, including scholarships, mentoring programs, financial education, and well-being resources. Mentorship, in particular, plays a crucial role in helping these students navigate the complexities of medical training and career development. Nguyen et al. highlighted the positive impact of mentorship on the academic success and well-being of first-generation medical students [11].

Future directions

Medical institutions and policymakers must recognize the unique contributions of first-generation students and invest in programs that foster their development. This investment is not only a moral imperative but also a strategic necessity for the future of the medical profession. By creating targeted programs that support first-generation students from the moment they express interest in medicine through their medical school journey and into their professional careers, we can help them overcome the obstacles they face. These programs should include mentorship opportunities, scholarships, academic advising, and mental health support tailored to the specific needs of first-generation students. Additionally, medical schools should actively recruit first-generation students, recognizing that their inclusion will bring valuable diversity to the medical profession. By doing so, we not only enrich the medical profession but also take a significant step towards creating a more equitable and inclusive healthcare system.

Conclusion

First-generation college students entering the field of medicine are invaluable assets to the healthcare system. These students bring unique and diverse perspectives shaped by their life experiences, which often include overcoming significant personal and societal challenges. Their journey to medical school is frequently marked by resilience, perseverance, and a strong commitment to serving underserved communities - qualities that are indispensable in the medical profession. The inclusion of first-generation students in medical education not only enriches the learning environment but also enhances patient care, as these students often have a deep understanding of the social determinants of health and a passionate dedication to addressing health disparities.

A healthcare system that truly represents the diversity of the population it serves is better equipped to meet the needs of all patients, particularly those from marginalized and underserved communities. First-generation students, with their unique perspectives and experiences, are crucial to this effort. They are more likely to practice in underserved areas and to work with populations that face significant health disparities. By supporting these students, we not only advance the cause of social justice in healthcare but also improve health outcomes for those who are most in need.

The future of medicine depends on the inclusion and success of these trailblazing individuals who represent the best of what our diverse society has to offer. As these students progress through their medical education and into their careers, they will bring with them a deep understanding of the challenges faced by underserved communities and a commitment to making a difference. Their success will inspire future generations of first-generation students to pursue careers in medicine, creating a positive cycle of inclusion and innovation in the healthcare system. By investing in the success of first-generation medical students, we are not only shaping the future of the medical profession but also building a more just and equitable society. These students are not just the future of medicine, they are the future of a healthcare system that is more compassionate, inclusive, and effective for everyone.
